# Reanalysis of genomic data in rare disease: current practice and attitudes among Australian clinical and laboratory genetics services

**DOI:** 10.1038/s41431-024-01633-8

**Published:** 2024-05-25

**Authors:** Stephanie Best, Zoe Fehlberg, Christopher Richards, Michael C. J. Quinn, Sebastian Lunke, Amanda B. Spurdle, Karin S. Kassahn, Chirag Patel, Danya F. Vears, Ilias Goranitis, Fiona Lynch, Alan Robertson, Emma Tudini, John Christodoulou, Hamish Scott, Julie McGaughran, Zornitza Stark

**Affiliations:** 1 Australian Genomics, Melbourne, VIC Australia; 2 University of Melbourne, Melbourne, VIC Australia; 3 Peter MacCallum Cancer Centre, Melbourne, VIC Australia; 4 Victorian Comprehensive Cancer Centre Alliance, Melbourne, VIC Australia; 5 Murdoch Children’s Research Institute, Melbourne, VIC Australia; 6 Centre for Population Genomics, Garvan Institute of Medical Research, University of New South Wales Sydney, Sydney, NSW Australia; 7 Centre for Population Genomics, Murdoch Children’s Research Institute, Melbourne, VIC Australia; 8 Genetic Health Queensland, Royal Brisbane and Women’s Hospital, Brisbane, QLD Australia; 9 Victorian Clinical Genetics Services, Murdoch Children’s Research Institute, Melbourne, VIC Australia; 10 Population Health Program, QIMR Berghofer Medical Research Institute, Brisbane, QLD Australia; 11 Adelaide Medical School, The University of Adelaide, Adelaide, SA Australia; 12 Department of Genetics and Molecular Pathology, SA Pathology, Adelaide, SA Australia; 13 The University of Queensland, Brisbane, QLD Australia; 14 The Genomic Institute, Department of Health, Queensland Government, Brisbane, QLD Australia; 15 Genetics and Molecular Pathology Research Laboratory, Centre for Cancer Biology, An alliance between SA Pathology and the University of South Australia, Adelaide, SA Australia

**Keywords:** Genetic testing, Genetic services

## Abstract

Reanalyzing stored genomic data over time is highly effective in increasing diagnostic yield in rare disease. Automation holds the promise of delivering the benefits of reanalysis at scale. Our study aimed to understand current reanalysis practices among Australian clinical and laboratory genetics services and explore attitudes towards large-scale automated re-analysis. We collected audit data regarding testing and reanalysis volumes, policies and procedures from all Australian diagnostic laboratories providing rare disease genomic testing. A genetic health professionals’ survey explored current practices, barriers to reanalysis, preferences and attitudes towards automation. Between 2018 and 2021, Australian diagnostic laboratories performed over 25,000 new genomic tests and 950 reanalyses, predominantly in response to clinician requests. Laboratory and clinical genetic health professionals (*N* = 134) identified workforce capacity as the principal barrier to reanalysis. No specific laboratory or clinical guidelines for genomic data reanalysis or policies were identified nationally. Perceptions of acceptability and feasibility of automating reanalysis were positive, with professionals emphasizing clinical and workflow benefits. In conclusion, there is a large and rapidly growing unmet need for reanalysis of existing genomic data. Beyond developing scalable automated reanalysis pipelines, leadership and policy are needed to successfully transform service delivery models and maximize clinical benefit.

## Introduction

Reanalyzing stored genomic data over time is highly effective in increasing diagnostic yield in rare disease. A recent systematic review and meta-analysis of 29 studies reported an overall diagnostic yield of 10% (95% CI = 6–13%), for individuals without a diagnosis following initial analysis, after an approximate median time of 24 months [1].

No universal definition of reanalysis exists, and the process can encompass a range of components, from re-phenotyping affected individuals, through to re-annotation of existing data, and re-prioritizing and re-evaluating variants [2]. The increase in diagnostic yield is currently driven by two major factors: improvements in knowledge about the genetic basis of rare disease and improvements in analysis processes. Commonly used curated databases of rare disease such as OMIM continue to add ~200 new entities annually [3]. At the variant level, interpretation improves over time with reports in the medical literature or in databases such as ClinVar, which now includes more than four million submitted variant interpretations. Conversely, the growth of reference population databases has repeatedly provided evidence against association of an increasing number of variants with rare disease [4, 5]. Re-curation of gene-disease relationships by expert groups within ClinGen can also refute diagnoses [6]. All these processes are important in improving not only diagnostic yield, but also the accuracy of diagnosis. From a bioinformatics perspective, realignment and reannotation of existing data, and integration of new and improved analysis for additional variant types such as short tandem repeats, copy number variants and other structural variants, also yield additional diagnoses.

Regular reanalysis of existing genomic data to improve diagnostic outcomes has been the subject of a “points to consider” statement by the American College of Medical Genomics and Genetics [7]. A position statement from the American Society of Human Genetics, endorsed by the Human Genetics Society of Australasia, emphasized the ethical obligation for diagnostic laboratories and research groups to support periodic reanalysis and return of results [8]. Preliminary data obtained in Australian cohorts [9, 10], including an assessment of cost effectiveness, formed the basis of a recommendation to include reanalysis as part of healthcare system funding via the Medicare Benefits Scheme for genomic testing in children with multiple congenital anomalies or syndromic and non-syndromic intellectual disability. As part of this determination, in Australia, two cycles of reanalysis are funded, to be performed on a case-by-case basis upon clinician request prior to the child reaching 15 years of age and after at least 18 months after the original test request [11]. The funding does not apply to reassessment of the previously reported variants of uncertain significance. In reaching this funding decision, the review process noted that automation is likely to replace the manual approach in the future, thus enabling more frequent reanalysis.

While the benefits of reanalysis are established and recognized at professional and health policy levels and automation is widely anticipated, reanalysis currently remains a heavily manual process. This, coupled with the shortage of a skilled genomics workforce and limited reimbursement, raises concerns that most existing datasets benefit from little if any reanalysis, creating an inequity in diagnostic, and by extension, health outcomes. Automation of the process will be essential to gain the full diagnostic benefits of reanalysis for existing and future rare disease genomic datasets, thus maximizing the value of investments in research and clinical sequencing. Several examples of automated approaches using open-source and commercial tools have already been published, with promising results [12–16].

Before commencing a national large-scale automated reanalysis research project, we sought to define baseline current clinical and laboratory practice in Australia, including reanalysis request volumes, guidelines, and barriers. In addition, we explored workforce attitudes towards automating the process.

## Methods

### Diagnostic genomic laboratory audit data

A quantitative audit data collection instrument was deployed in February 2022 via the online research platform REDCap to the heads of all Australian diagnostic laboratories accredited to provide rare disease genomic testing (exome or genome). Data were collected regarding genomic test volumes from 2018 to 2021 performed on exome and genome backbones, including large virtual panels (defined as those >100 genes). Data were also collected on reanalyses performed during the same period, either laboratory- or clinician-initiated, and on laboratory reanalysis guidelines and policies. Laboratories were also asked to supply the clinical genomic consent forms currently in use. A copy of the audit instrument can be found in Supplementary information.

### Workforce survey

#### Distribution

Clinical and laboratory genetic professionals (including trainees) working in rare disease in Australia were invited to participate via the mailing lists of the Human Genetics Society of Australasia and Australian Genomics between February and April 2022.

Participants were asked to complete the survey after reading a plain language statement and considered eligible if they were, or were training to be, a clinical geneticist, genetic counselor, clinical scientist, bioinformatician or genetic pathologist working in rare disease diagnostics in Australia. Participants had the option of providing contact details for follow-up focus groups and to receive a copy of the results.

#### Survey design

Survey questions were developed in consultation with an expert working group, comprising clinical geneticists, laboratory scientists and health services researchers (CR, ZF, MQ, DP, SL, ABS, KK, CP, DV, IG, FL, AR, ET, JS, HS, JM, SB, ZS).

The survey included categorical and free-text response options and was administered via REDCap, with an estimated time to completion of 10–15 min. A copy of the survey instrument can be found in Supplementary information. Participants in the workforce survey were divided into three groups based on their primary position: (i) “clinical respondents” including clinical geneticists and genetic counselors, including trainees; (ii) “laboratory respondents” including clinical scientists, bioinformaticians and genetic pathologists and trainees; (iii) “other respondents” including those that indicated a primary position other than those provided (e.g., academics or physicians with multiple qualifications including clinical genetics).

In brief, those who identified as clinical respondents were asked questions related to their own current practice and reanalysis requests in the last 12 months, including how this was funded, and whether guidelines and policies were in place at their clinical service. Clinical and laboratory respondents were additionally asked to rank barriers to reanalysis in the current system and select an ideal frequency for reanalysis to be conducted on unsolved cases if the current barriers were resolved.

After reading a description of a proposed automated model for the reanalysis of genomic data, all respondents were asked to complete validated Likert-scale questions regarding two implementation outcomes of acceptability and feasibility which are thought to be prominent pre-implementation [17]. Acceptability items were generated through a combination of the Acceptability of an Intervention Measure (AIM) [18] and the Theoretical Framework of Acceptability (TFA) [19, 20]. Feasibility items aligned with the Feasibility of Intervention Measure (FIM) [18] (Supplementary Table 1). A free text space for respondents to detail perceived benefits and barriers to an automated approach to reanalysis was provided.

#### Survey analysis

Data extracted from the surveys were analyzed using descriptive statistics to summarize demographics and frequency of other survey responses. Where appropriate, survey responses were compared between clinical and laboratory respondents. During analysis of the acceptability and feasibility questions, the five-point Likert-scale collapsed into three categories (disagree, neither agree nor disagree, and agree) and the items related to the AIM and FIM scales were combined to provide an overall score. The TFA tool provides a more granular look at acceptability with those scores analyzed and presented separately. Negatively worded Likert scale items were reversed scored during analysis.

Qualitative data generated from the free-text comments section was analyzed using conventional content analysis to identify recurrent themes [21]. Two researchers reviewed the data (ZF & SB) and themes were identified and refined during weekly meetings. A third researcher (ZS) was available to support decision-making where agreement was not reached [22].

## Results

### Australian clinical genomics laboratories: diagnostic and reanalysis test volumes in rare disease

All seven Australian diagnostic laboratories currently accredited to provide genomic testing in rare disease provided audit data on genomic test volumes and reanalysis requests from 2018 to 2021, Fig. 1. Large virtual panels were the commonest test type in 2018 but have now been surpassed by exome and genome test requests. The overall volume of genomic tests in rare disease has grown by 55% in the past 4 years, while reanalysis requests have remained at <5% of new test requests.

**Fig. 1:**
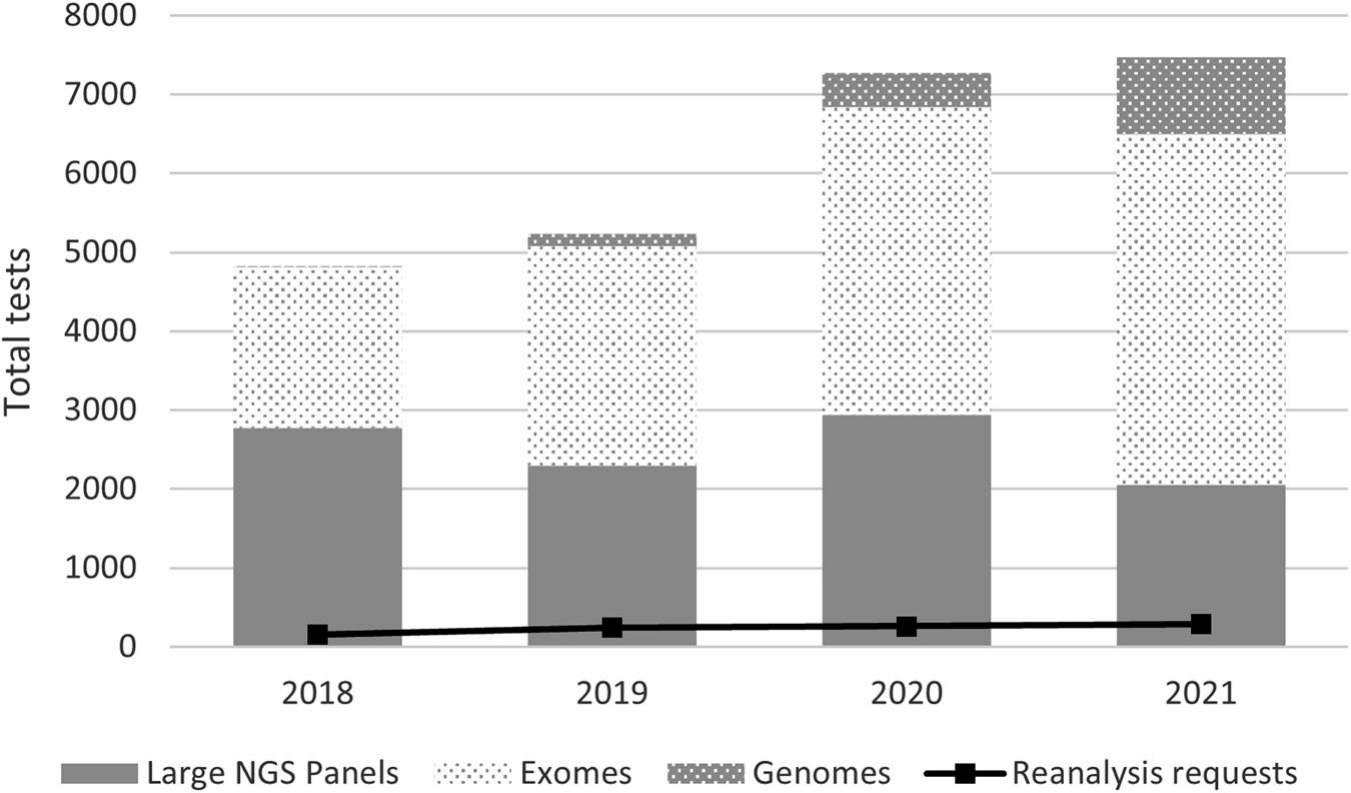
Aggregate genomic tests and reanalysis requests from Australian clinical genomics laboratories currently accredited to provide genomic testing in rare disease from 2018 to 2021. For each year, a breakdown of primary test requests and type (large NGS panel, exome, genome) and of reanalysis requests is provided.

### Australian clinical genomics laboratories: current reanalysis policies and procedures

No laboratories reported having formal and specific guidelines or policies regarding reanalysis. Two laboratories indicated that guidelines for reanalysis were addressed within other policy documents and primarily focused on practical aspects of managing reanalysis requests.

Diagnostic laboratories reported primarily performing reanalysis based on individual clinical requests. Four of seven (57%) of laboratories reported having performed laboratory-initiated reanalysis due to updates in bioinformatics pipelines, updates to variant interpretation knowledge databases used, and rectifying systematic errors.

All laboratories accepted reanalysis requests by the original referring clinician or other clinicians involved in patient care. Two laboratories indicated that reanalysis requests can be made by researchers and one by genetic pathologists. No laboratories reported accepting reanalysis requests directly from affected individuals or families.

Three laboratories did not accept any reanalysis requests for externally generated data, while four accepted data from other Australian clinically accredited laboratories, three from overseas accredited laboratories, and three from research studies.

The fee for reanalysis varied between laboratories, ranging from AUD$200 (USD$127, E120) to AUD$425 (USD$270, E256).

### Consent form analysis

Seven consent forms currently in use were sourced from diagnostic laboratories. Of these, two were directly based on the national clinical consent form for genomic testing template developed by Australian Genomics, and two were modeled on the template (https://www.australiangenomics.org.au/tools-and-resources/national-clinical-consent-forms/). None of the consent forms included specific clauses for reanalysis. However, all consent forms included statements about the possibility of future testing or analysis (e.g., “The sample may be examined again in the future using new methods/technologies”). The Australian Genomics information sheet which is designed to accompany the consent form does not specifically refer to reanalysis of genomic data. However, it states that “*if a diagnosis is not found today, the genomic test result could be looked at in the future as our understanding improves*”.

### Clinical and laboratory workforce survey: demographics

A total of 185 eligible participants responded to the survey, of whom 51 (28%) did not proceed satisfactorily and were excluded. This resulted in 134 responses that were suitable for analysis, of which 124/134 (93%) fully completed the survey. The remaining respondents completed a subset of questions and were included in the analysis for the questions they answered. Because of the broad dissemination approaches used by professionals working in both rare diseases and cancer, as well as those working outside of Australia, we are unable to calculate an accurate response rate. Of the 134 survey participants, 84 (63%) were clinical (37 clinical geneticists, 27.6%; 47 genetic counselors, 35.1%). There were 38 laboratory respondents (28%), including 28 clinical scientists (21%), two bioinformaticians (2%) and eight genetic pathologists (6%).

### Workforce survey: current clinical practice

A third of clinical respondents (29/84, 35%) reported ordering between 20 and 50 genomic tests in the last year, with 24% (20/84) ordering less than 10, and 23% (19/84) ordering more than 50. A third (33%, 28/84) had not ordered any reanalysis in the past year, 42% (35/84) had ordered reanalysis less than 5 times, 17/84 (20%) between 5 to 10 times, 3/84 (4%) between 10 to 20 times and 1/84 (1%) more than 20 times.

The top-ranked reason for ordering reanalysis was passage of time in an undiagnosed individual (relative score = 1), followed by new clinical information (relative score = 0.88), referral back for reassessment (relative score = 0.85), awareness of new gene discoveries relating to the phenotype (relative score = 0.74), family request (relative score =0.51) and awareness of change in laboratory processes (relative score = 0.49).

For those clinical respondents who provided estimates of where they send their genetic testing (*n* = 81), the highest proportion was sent to their local state-based laboratories (mean of estimates (*M*) = 45%, standard deviation (SD) = 34.4), followed by overseas laboratories (*M* = 24%, SD = 27.3), interstate laboratories (*M* = 17%, SD = 20.57) and research studies (*M* = 12%, SD = 24.85).

Most clinicians (*n* = 67, 81%) reported their clinical service did not have written guidelines or policies about reanalysis, 13 (16%) were unsure and 3 (4%) reported the existence of clinical guidelines. Clinicians reported most reanalysis requests are currently funded through clinical genetics services budgets (56, 67%) or hospital budgets (30, 36%), with a minority reporting funding by laboratories (9, 11%) or other sources such as families and research studies (7, 8%).

Just over half of clinicians (44, 52%) were aware of government-approved funding for reanalysis through the Medicare Benefits Scheme for individuals with syndromic and non-syndromic ID, which become effective May 2022, with 37 (44%) not aware of this and 3 (4%) unsure.

### Workforce survey: current barriers to reanalysis of genomic data in rare disease

**Fig. 2:**
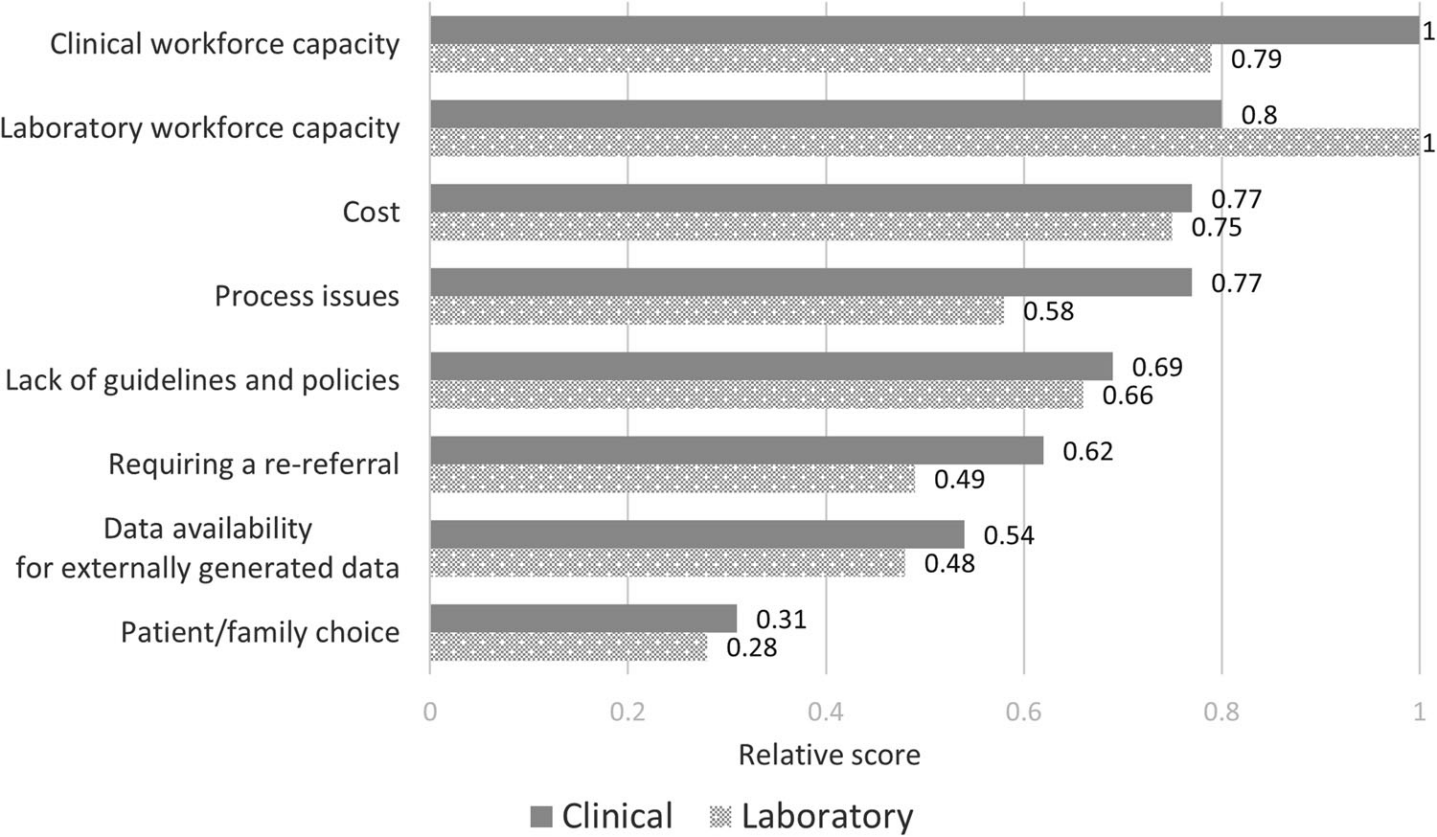
Ranking of current barriers to the reanalysis genomic data for unsolved rare disease cases from clinical (*n* = 82) and laboratory (*n* = 35) survey respondents. For each barrier, the relative score assigned by clinical and laboratory respondents is shown.

All survey respondents were asked to rank, from highest to lowest, the current barriers to reanalysis based on their experience from the list of reasons provided (Fig. 2). Workforce capacity was the highest ranked barrier overall and for each group respectively (relative score = 1), followed by cost (relative scores, clinical = 0.77 laboratory = 0.75) and process issues (relative scores, clinical = 0.77 and laboratory = 0.58).

### Workforce survey: preferences for the frequency of reanalysis for unsolved rare disease cases

**Fig. 3:**
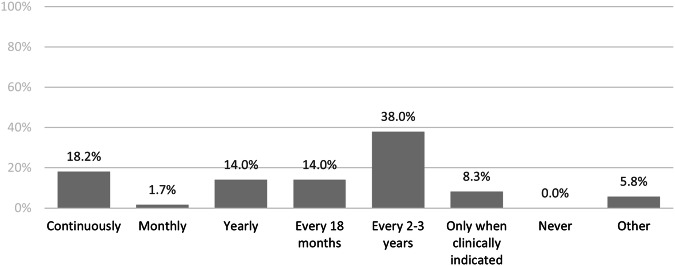
Preferences for the frequency of reanalysis for unsolved rare disease cases if current barriers were resolved from clinical and laboratory respondents (*n* = 121). Proportion of respondents favoring different reanalysis intervals ranging from ‘continuous’ to ‘never’ shown.

Assuming the current barriers were resolved, there was a range of preferences regarding optimum frequency (Fig. 3). While 2–3 yearly reanalysis was the most favored option 46/121 (38%), earlier reanalysis was favored by 58/121 (48%), with 22/121 (18%) professionals preferring continuous reanalysis.

The reasoning behind these preferences was expanded on in the free text comments (*n* = 88). Some respondents felt a clinically indicated approach mitigates challenges to recontacting individuals/families, ensuring consent, integrating new clinical information, and the ability to triage those who would benefit most from reanalysis. Respondents reasoned that reanalysis that occurs every 2–3 years’ time would be cost-effective and balance new disease gene discoveries and technology advances with clinical and laboratory workloads. One respondent also considered the timeframe acceptable to individuals and families. Example: *It provides a concrete timeframe which patients appreciate, a reasonable amount of time for new information to become available but is not a burden on the patient or the health service*. (Clinical staff #31). Likewise, respondents described that every 18 months or yearly would provide adequate time for new literature to arise without overwhelming laboratory or clinical services. Example: *This seems to be the goldilocks [zone] between gene discovery and not overloading the clinical/lab services*. (Clinical staff #10).

A continuous approach was perceived ideal by some given the unpredictability of when a new gene-disease discovery may be made and the ensuing implications of new information for individuals/families, especially for reproductive planning. Example: *If there was an ability to automatically review and flag cases against new information, ideally, we would want to know about this new information as soon as it becomes available*. (Laboratory staff #5).

Other respondents reasoned different timeframes should be applied depending on individual/family circumstances or likelihood of a diagnostic yield. Example: *There should be levels of requests for re-analysis according to patient need/clinical urgency for example, a family where a genetic diagnosis implies reproductive confidence should be in “ continuously” category while those where it less critical could be in the 2–3 yearly category* (Clinical staff #146). Others noted options for quarterly, bi-annually or 5-yearly reanalysis.

### Workforce Survey: perceptions of acceptability and feasibility towards automated reanalysis of genomic data in rare disease

**Fig. 4:**
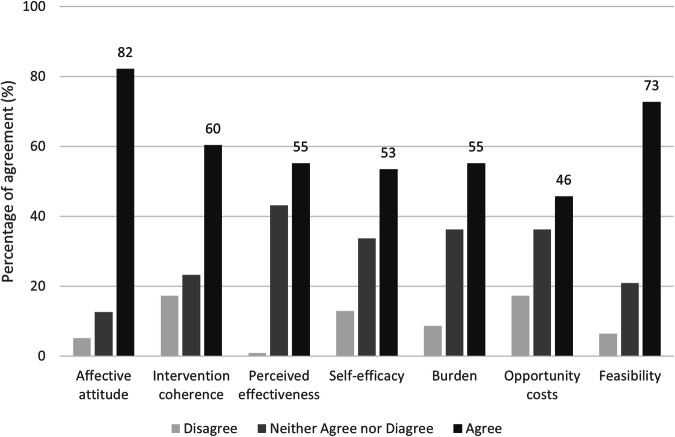
Acceptability (six outcomes) and Feasibility agreement fractions from clinical and laboratory survey respondents (*n* = 116). For each outcome, percentage of agreement among respondents is shown.

After reading a description of a proposed automated model for the reanalysis of genomic data, 116 respondents (80 clinical and 36 laboratory) completed validated measures of acceptability and feasibility [18, 20]. Overall, there was little difference observed between clinical and laboratory responses (Supplementary Fig. 1). Acceptability regarding affective attitude i.e., how people feel about automating reanalysis of genomic data of the proposed automated model was considered as highly agreeable (82%) and somewhat feasible (73%). Less agreeable were acceptability outcomes related to intervention coherence i.e., the extent to which participants understand automating reanalysis of genomic data and how it would work (60%), the perceived effectiveness (55%), self-efficacy to carry out automated reanalysis (53%), the perceived burden (55%) and opportunity costs (46%) (Fig. 4).

### Workforce survey: perceived benefits and barriers towards automated reanalysis of genomic data in rare disease

Seventy-one respondents (57%) (52 clinical and 19 laboratory) gave a written response to their perceived benefits and barriers to the automated reanalysis program. Table 1 provides an overview of the benefits and barriers alongside exemplar responses.

**Table 1:** Clinical and laboratory staff’s perceived benefits and barriers to adopting an automated approach to reanalysis of genomic data in rare disease with example responses.

Benefit	# of comments	Example
Patient benefits including timely diagnosis, improving disease treatment or management, information for reproductive planning, reducing barriers to access and improving patient experience of genetics services	17	*Benefits for the patient’s management if the cause is found—new variants would benefit the family planning and reproductive choices*… *Improving clients/families experience and satisfaction by the clinical genetic service*. (Clinical staff #66)
Workforce benefits of a streamlined and efficient process, lessen the burden on HCPs to remember to prompt re-referral and a sense of satisfaction with maximizing current knowledge	19	*Less manual input required for re-analysis so less strain potentially on already stretched lab resources*. (Clinical staff #202)*Does not put the onus back on the referring doctor to remember to re-refer*. (Clinical staff #42) *Leveraging on new discoveries to provide more diagnoses and once implemented, relatively low cost and low manual labor* (Laboratory staff #89)
Barrier		
Increased clinical workloads, re-contacting families, advice on reportable findings	15	*Increased workload for clinicians/clinical services to see families once results of reanalysis are available (although improvement in terms of not needing to see all patients to organize reanalysis)*. (Clinical staff #71) *Increased workload if it requires further clinical/MDT discussion/evaluation of newly found variants*. (Clinical staff #66)
Returning results to referring clinicians and patients/families, including re-contacting families with outdated contact info, ethical/legal implications and increasing patient anxiety	13	*Logistics of contacting and discussing unexpected (due to timing) results with family can be difficult*. (Clinical staff #132) *Communicating results from lab to patient (via referrers who may have moved on)*. (Laboratory staff #47)
Increased laboratory workloads and implications for turn-around-times of other diagnostic tests	7	*The workforce capacity issues of re-evaluation of flagged variants and subsequent re-issue of reports*. (Laboratory staff #14)
Data availability, uniformity, and quality of the metadata.	7	*Interoperability with different sources of laboratory data; consent; privacy; data security; cost*. (Laboratory staff #144)
Funding	6	*The only major barrier I see is issues with funding and time allocation*. (Clinical staff #176)
Laboratory or clinical staff willingness to adopt change	5	*Barriers I believe will be the required shift of mindset surrounding this topic. Part of this is the claim that we do not have the resources (and yet our role in genetics includes helping people navigate their genetic odyssey so somehow we need to “make the time/resources”!). The other part is making assumptions on the patients’ behalf about what “they need to know”. Patients are resilient and intelligent beings who deserve access to information to help on their journey - and we can support them in their understanding and adjustment to new information*. (Clinical staff #20) *Major hurdle will be funding, data availability and laboratory willingness to participate*. (Clinical staff #26)
Returning incidental findings to patients/families	4	*Potentially having to provide patients with many VUS results or incidental findings*. (Clinical staff #102)
Development of laboratory software and processes	4	*Development of a variant analysis model that yields accurate classifications and a low number of false positives*. (Laboratory staff #89)
Consistency of service delivery	3	*Barriers are ensuring consistent care across services of communicating reanalysis results to families*. (Clinical staff #13)
Distrust in the process	3	*Lack of trust in system i.e., what if the automated analysis misses something, change in family circumstances that we may not be aware of - meaning they may no longer want results*. (Clinical staff #63)
Shortage of skilled laboratory staff	2	*Adequate skilled curation scientists*. (Laboratory staff #151)

Respondents described the clinical benefits as finding a timely diagnosis, improving disease treatment or management, information for reproductive planning, reducing barriers to access and improving individual and family experience of genetics services. Clinical or laboratory staff acknowledged a streamlined automated process would likely reduce clinical and laboratory workloads, lessen the burden on clinical staff to remember to prompt re-referral, and produce a sense of satisfaction with maximizing current knowledge.

The most widely anticipated barrier was increases to clinical staff workloads regarding return of results and providing input on potential new findings. Additional challenges to results return included the requirement for re-contact especially if contact information is out-of-date, and the subsequent legal/ethical implications for clinicians. Respondents raised concerns over increasing anxiety around unknown timing of return of results. Challenges associated with ensuring informed consent were frequently raised with additional concern perceived around transition between pediatric and adult services. Funding barriers were also tied to increased workforce demands within what was described as an already under-resourced setting. Increases to laboratory staff workloads was less of a concern, however a shortage of skilled clinical scientists was noted. Laboratory staff barriers centered more on the availability of data including accessing uniform metadata or sharing data between laboratories. Further staff barriers included possible mistrust towards new technologies and a perceived unwillingness from within the wider laboratory and clinical community to adopt the new approach.

## Discussion

National data collected from Australian diagnostic laboratories reveals a large, rapidly growing unmet need for reanalysis of existing datasets. Only considering those tested between 2018 and 2021, we estimate that 17,000 Australians with rare disease would benefit from reanalysis (assuming a conservative 30% diagnostic yield from the initial analysis). However, only 950 (5%) have been reanalyzed to date, demonstrating that the current, heavily manual process of reanalysis benefits a very small proportion of families and health services. These data are consistent with audit data of federally-funded test requests in Australia, which revealed marked underutilization, with only 12 reanalysis requests being funded through this pathway in the first year of operation [23]. As testing volumes increase, this gap is only likely to grow under the current model.

Our data raise concerns not only about the low volume of reanalysis overall but also about inequity of access and ultimately, inequity of outcomes [24]. Currently, federal funding for reanalysis in Australia is not only underutilized but is also limited to a small subset of individuals under the age of 15 years, with two cycles funded [11]. Reanalysis requests outside of these restrictive eligibility criteria are funded through a variety of sources without any policies or guidelines in place, leaving access to be determined by individual clinicians and services. Of note, individual practice was highly variable with a third of clinicians having not requested any reanalysis in the last year. This lack of consistency is reflected in studies of the experience of affected individuals and families who report being left without clear communication regarding the timing, initiation, and process of reanalysis [25]. Further inequities arise at laboratory level with a large proportion of genomic tests in Australia being performed through research studies, overseas and interstate laboratories and yet three of seven laboratories only accepting reanalysis requests on internally generated data. Those that do accept requests on externally generated data face additional barriers including managing data access, transfer, and reprocessing. These issues highlight the need for nationally cohesive approaches and genomic data management systems that facilitate reanalysis of clinically as well as research-generated genomic data.

Genetic professionals identified workforce capacity as the principal barrier to reanalysis under the current model. Automation has been highlighted as a possible solution to this key issue [14]. There was general concordance between clinical and laboratory staff perceptions of acceptability and feasibility of automated approaches. Overall, respondents personally considered an automated approach to analysis as highly acceptable. This finding supports the anticipated clinical and workforce benefits and challenges the idea raised by some that there may be an unwillingness within the broader workforce to adopt automation. Interestingly, other aspects of acceptability garnered by using a more granular tool [19] showed lower levels of acceptability around opportunity costs and burden, which is in keeping with previous research [26] and increased workloads as the most salient barrier reported. The low acceptability scores around self-efficacy, perceived effectiveness and intervention coherence may be explained given these questions were asked pre-implementation and would be worth repeated post-implementation to observe changes [27]. Whilst an automated approach was considered somewhat feasible, possible explanations as to reservations may relate to barriers raised around the logistics of consent, return of results, and funding. Despite having high personal acceptability indicating a motivated clinical and laboratory workforce who welcome automation, our findings suggest that barriers around logistics and feasibility of working within resource-constrained healthcare settings likely overshadows the former. Understanding the cost implications of automated models and designing appropriate funding pathways will be an important component of sustainable implementation.

This study has limitations. While the qualitative data from this study adds to our understanding of the barriers to reanalysis of genomic data, a more nuanced understanding of the workforce issues raised by reanalysis and automation may be gained through in-depth qualitative approaches, such as process mapping [28] and focus groups with health professionals. This study focused on the views of those delivering reanalysis of genomic data. Ascertaining the views of individuals and families affected by rare disease will be equally important in guiding policy and practice, including the design of service delivery models [29].

Our study reveals a large and rapidly growing unmet need for reanalysis of existing clinical genomic data as well as lack of policies and highly variable clinical and laboratory practices, raising significant concerns about inequity of access. Beyond demonstrating the performance of automated systems in large-scale studies, leadership at multiple levels including clinical and laboratory services, as well as policy makers will be required to successfully transform service delivery models and maximize the benefits of automation for both health professionals and families affected by rare disease.

## Supplementary information

Supplementary material

## Data Availability

Data are available from the authors upon individual request. Privacy guidelines and/or regulations may limit or restrict sharing to certain individuals, such as those trained in Human Subjects Research and privacy protections.
