# Supplementary material for: Reanalysis of genomic data in rare disease: current practice and attitudes among Australian clinical and laboratory genetics services
Source: Eur J Hum Genet. 2024 May 25;32(11):1428–35. doi: 10.1038/s41431-024-01633-8 (PMC11576731; doi:10.1038/s41431-024-01633-8)
Supplement: Supplementary file 1 — Supplementary material [file 41431_2024_1633_MOESM1_ESM.docx]

Supplementary Information 1: Laboratory Audit Instrument

Definitions: Reanalysis and other terms in this survey may have different meanings for different groups depending on their context. When responding to the questions in this survey, please do so with the following in mind:

For the purposes of this survey, reanalysis is defined as the process of re-examining existing genomic data from an individual. This is an overarching term that may encompass all of the definitions and concepts outlined in the table below. For the purposes of this survey, a genomic test includes whole genome, whole exome and large next-generation panel sequencing (those containing more than 100 genes)

**Survey of Australian clinical genetic testing laboratories reanalysis practices**

| Which laboratory/group do you represent? |  |
| --- | --- |
| For each year listed, please indicate the approximate number of rare disease cases that had Large NGS Panels (greater than 100 genes), Exomes and Genomes analysed by your laboratory:  Note: please consider each proband as one case (i.e. a trio counts as one case) | Large NGS Panels (greater than 100 genes):  DO NOT INCLUDE VIRTUAL PANELS DONE ON EXOME OR GENOME BACKBONE  2018 2019 2020 2021  ______ ______ ______ ______  Exomes  2018 2019 2020 2021  ______ ______ ______ ______  Genomes  2018 2019 2020 2021  ______ ______ ______ ______ |
| For each year listed, please indicate the approximate number of formal, clinician-initiated renanalysis requests conducted in your laboratory.  Formal requests are those that occur via an established pathway (i.e. via a formal request form) as opposed to informal requests such as casual emails/phone calls. | 2018 2019 2020 2021  ______ ______ ______ ______ |
| In the last 3 years, have there been any circumstances under which your laboratory has conducted laboratory-initiated reanalysis? | {1} Yes {2} No |
| Please list under which circumstances (i.e. what was  the trigger for conducting laboratory-initiated reanalysis) | {1} Updates to bioinformatics pipelines  {2} Updates to variant interpretation knowledge  {3} Updates to gene disease knowledge databases (e.g PanelApp, GenCC)  {4} Updates to population and disease allele  frequency databases (e.g. gnomAD)  {5} Updates to functional and computational data  tools (e.g. new insilico predictors)  {6} Rectifying systematic errors that are  identified  {7} Other |
| Please list other circumstances |  |
| laboratory currently accepts. |  |
| Are you willing to share an example of the consent  form(s) you accept? All information will be kept  confidential and used for thematic analysis. If  willing, please attach. Any patient information on  example consent forms should be redacted.  If possible, please attach all forms as a single file. | {1} Yes {2} No |
| Does your laboratory have any written or published  guidelines or policies in place regarding reanalysis? | {1} Yes {2} No |
| Are you willing to share your guidelines or policies  regarding reanalysis? We will use these for thematic  analysis and all information will be kept confidential.  If possible, please upload all document as a single  file. | {1} Yes {2} No |
| Who can request reanalysis through your laboratory? | {1} Original referring clinician  {2} Other clinicians involved in patient care  {3} Patients/families  {4} Other |
| Please list other requestors. |  |
| Who do you issue reanalysis reports to in your laboratory? Check all that apply | {1} Original referring clinician laboratory?  {2} Requesting clinician (if not original)  {3} Other |
| Please list other recipients |  |
| Does your laboratory accept reanalysis requests for  externally generated data? Check all that apply. | {1} Yes, from Australian NATA-accredited  externally generated data?  {2} Yes, from overseas accredited laboratories  {3} Yes, from research studies  {4} No  {5} Other |
| Please list source of reanalysis request. |  |
| In your laboratory, what is the current cost for  Reanalysis? |  |

Supplementary Information 2: Workforce Survey Instrument

**Genomic data and reanalysis: what are your current practices and preferences?**

| **Characteristics of respondents** | |
| --- | --- |
| What is your primary position?  If you're still training, please select the relevant field | {1} Clinical geneticist  {2} Genetic counsellor  {4} Clinical scientist professional group  {5} Bioinformatician  {6} Genetic pathologist  {7} Other |
| What is your primary position? |  |
| How many years have you been working in clinical/laboratory genetics? | {1} < 5  {2} 6-10  {3} 11-15  {4} 16-20  {5} 21-15  {6} >25 |
| Which state/territory do you work in? Check all that apply. | 1} New South Wales  {2} Queensland  {3} Northern Territory  {4} Western Australia  {5} South Australia  {6} Victoria  {7} Australian Capital Territory  {8} Tasmania |
| **Frequency and reasons for genomic testing and reanalysis** | |
| How many genomic tests have you ordered in the last year? Note: for this question, a genomic test includes whole genome, whole exome and large next-generation panel sequencing (e.g. those containing more than 100 genes). | {1} < 10  {2} 10-20  {3} 20-50  {4} >50 |
| In the last year, please indicate the approximate proportion (in percentage) of your genomic testing that goes to: | State-based laboratory, Interstate laboratory Overseas laboratory, Research Study |
| How many times in the last year have you requested reanalysis for your rare diseases patients? Note: reanalysis is defined as the process of re-examining existing genomic data from an individual. | {1} Not in last year  {2} < 5  3} 5-10  {4} 10-20  {5} >20 |
| When reanalysis is requested, what are your reasons for doing so? Please rank from the most common (1) to least common (6) reasons from the list provided.  If you have additional reasons not on the list or you would like to expand on your rankings, please do so in the space provided. | - New clinical information about the patient - Passage of time in undiagnosed patient - Family request - Awareness of change in laboratory processes, e.g. bioinformatic pipeline - Awareness of changes in discovered genes relating to patient phenotype e.g. updated gene panel content - Patient referred back for reassessment |
| **Guidelines** | |
| Does your clinical service have any written guidelines or policies in place about when reanalysis should be performed? | {1} Yes {2} No {3} Unsure |
| Would you be willing to share the guideline or policy you use? All information will be kept confidential and used for thematic analysis. If willing, please attach. |  |
| If you would like to provide additional comments about your answer, please do so in the space provided. |  |
| **Funding** | |
| How are reanalysis requests funded in your clinical practice? Check all that apply.  If there are other sources of funding, please specify. | {1} Clinical service budget  {2} Hospital-based budget  {3} Family pays  {4} Laboratory covers the cost  {5} Other |
| **Medicare Benefits Scheme number awareness** | |
| Are you aware of the MBS reanalysis item 73360? See here for details. | {1} Yes {2} No {3} Unsure |
| Do you have any comments regarding the conditions attached to this item or its practical implementation |  |
| **Current Barriers to reanalysis** | |
| In your experience, what are the current barriers to reanalysis? Please rank from the most common (1) to least common (8) from the list provided.  If you have additional barriers not on the list or you would like to expand on your rankings, please do so in the space provided. | - Cost - Process issues - Clinical workforce capacity e.g. appointment availability/workload - Laboratory workforce capacity e.g. reviewing reanalysis outputs and curation - Lack of guidelines and policies - Requiring a re-referral - Patient/family choice - Data availability for externally generated data |
| If current barriers were not an issue, how often do you think data from unsolved cases should ideally be reanalysed? | {1} Continuously  {2} Monthly  {3} Yearly  {4} Every 18 months  {5} Every 2-3 years  {6} Only when clinically indicated  {7} Never  {8} Other |
| Please explain the reasoning for your response or indicate another time period and your reasoning for choosing this. |  |
| **Feasibility and Acceptability of the proposed automated reanalysis approach** | |
| Please read about the GHFM reanalysis program rationale, description and key considerations below. GHFM program rationale Reanalysis of existing genomic data has repeatedly been shown to deliver additional yields in the range of 10-15%. Despite the well-established and accepted benefits, translation into practice is currently limited by reliance on a heavily manual process that is not scalable. Our program will harness continuously updated knowledge bases of disease-associated variants and genes, improvements in genomic data analysis and interpretation, and the use of cloud-based distributed systems supplemented with machine learning approaches to scale up and establish a national automated reanalysis program to increase rare disease diagnoses. GHFM program description Genomic data from unsolved cases will be re-aligned, re-annotated and continuously interrogated to identify likely pathogenic and pathogenic variants related to the original reason for testing, for example as a result of newly published gene-disease associations or identification of new variant types, such as small deletions or short tandem repeats. This is expected to benefit thousands of patients that do not have a diagnosis. Key considerations This reanalysis pipeline will be designed so that negative cases are automatically reanalysed in an iterative cycle without requiring clinician-initiated requests. Highly suspicious variants flagged by the automated reanalysis program will be formally assessed by diagnostic laboratories and pathogenic/likely pathogenic findings will be reported to the original requesting clinicians. Please answer the following questions in relation to the above described automated reanalysis program. These questions aim to understand the feasibility and acceptability of the proposed program. | |
| Please answer the following questions in relation to the above described automated reanalysis program. These questions aim to understand the feasibility and acceptability of the proposed program. |  |
| *Acceptability (affective attitude)*  The automated reanalysis program meets my approval.  The automated reanalysis program is appealing to me.  I like the automated reanalysis program.  I welcome the automated reanalysis program.  The automated reanalysis program aligns with my values and beliefs.  *Acceptability (logistics)*  I understand how the automated reanalysis program will work.  The automated reanalysis program will achieve its purpose.  I am confident that I can deliver my element of the automated reanalysis program.  It would take too much effort to participate in the automated reanalysis program.  I would have to give up other activities (I value) to participate in the automated reanalysis program.  *Feasibility*  The automated reanalysis program seems implementable.  The automated reanalysis program seems possible.  The automated reanalysis program seems doable.  The automated reanalysis program seems easy to use | {1} Completely disagree  {2} Disagree  {3} Neither agree nor disagree  {4} Agree  {5} Completely agree |
| **Benefits and barriers to automated reanalysis** | |
| Based on the description above, what do you see as the major benefits and barriers regarding the implementation of the program? |  |

Supplementary Table 1: Acceptability and Feasibility survey questions and corresponding measurement tool

| Acceptability (affective attitude) (AIM) | The reanalysis project meets my approval. |
| --- | --- |
|  | The reanalysis project is appealing to me. |
|  | I like the reanalysis project. |
|  | I welcome the reanalysis project. |
|  | The reanalysis project aligns with my values and beliefs. |
| Acceptability (TFA) | Intervention coherence: I understand how the reanalysis project will work. |
|  | Perceived effectiveness: The reanalysis project will achieve its purpose. |
|  | Self-efficacy: I am confident that I can deliver my element of the reanalysis project. |
|  | Burden: It would take too much effort to participate in the reanalysis project. |
|  | Opportunity costs: I would have to give up other activities (I value) to participate in the reanalysis project. |
| Feasibility (FIM) | The reanalysis project seems implementable. |
|  | The reanalysis project seems possible. |
|  | The reanalysis project seems doable. |
|  | The reanalysis project seems easy to use. |

Supplementary Figure 1: Breakdown of acceptability and feasibility questions by clinical and laboratory respondents
